# Effects of Dietary Yogurt on the Healthy Human Gastrointestinal (GI) Microbiome

**DOI:** 10.3390/microorganisms5010006

**Published:** 2017-02-15

**Authors:** Daniel J. Lisko, G. Patricia Johnston, Carl G. Johnston

**Affiliations:** Biology Department, Youngstown State University, Youngstown, OH 44555, USA; djlisko01@gmail.com (D.J.L.); gpjohnston@ysu.edu (G.P.J.)

**Keywords:** 16S rDNA, gut microbiome, yogurt, diversity, next generation sequencing

## Abstract

The gastrointestinal (GI) tract performs key functions that regulate the relationship between the host and the microbiota. Research has shown numerous benefits of probiotic intake in the modulation of immune responses and human metabolic processes. However, unfavorable attention has been paid to temporal changes of the microbial composition and diversity of the GI tract. This study aimed to investigate the effects of yogurt consumption on the GI microbiome bacteria community composition, structure and diversity during and after a short-term period (42 days). We used a multi-approach combining classical fingerprinting techniques (T-RFLPs), Sanger analyses and Illumina MiSeq 16S rRNA gene amplicon sequencing to elucidate bacterial communities and *Lactobacilli* and *Bifidobacteria* populations within healthy adults that consume high doses of yogurt daily. Results indicated that overall GI microbial community and diversity was method-dependent, yet we found individual specific changes in bacterial composition and structure in healthy subjects that consumed high doses of yogurt throughout the study.

## 1. Introduction

The human microbiome is the ecological community of commensal and symbiotic microbes and their genomes that populate every surface of the human body. The microbiome was previously estimated to be composed of nearly 100 trillion microbial cells that outnumber human cells in a 10:1 ratio [1]. This ratio was recently updated and more accurately determined to be within an order of magnitude (closer to ~1:1) using determinations of both microbiome cell numbers (~38 trillion) and human cell numbers (30 trillion cells) based on a 70 kg “reference” human containing a total microbiome mass of 200 g [2]. The gastrointestinal tract (GI) encloses the gut microbiome, which can include long-term or short-term resident bacteria acquired through diet [3]. The gut microbiome is not homogenous; both microbial density and diversity increase distally through the GI tract towards the colon, which contains roughly 10^11^–10^12^ bacteria cells per gram of luminal content [4]. Thus, fecal samples are most often used to monitor human GI microbiota since sampling is noninvasive [5,6]. The human gut is primarily dominated by *Bacteroidetes* and *Firmicutes*, including *Bifidobacteria*, *Lactobacillus*, *Streptococcous*, and *Clostridia* [1], whereas *Proteobacteria*, *Verrumicrobia*, *Actinobacteria*, *Fusobacteria*, and *Cyanobacteria* have been found in lesser proportions [7]. 

Over the past decade, the use of probiotics has generated renewed interest as a potential method for prevention of multiple gastrointestinal diseases, as well as for providing health benefits to the host [8] by modulating immunological responses [9,10]. Commensal microbiota, such as *Bifidobacterium* and *Lactobacillus*, both found in yogurt, have been traditionally consumed for their numerous potential health benefits. There are a number of studies indicating the positive effects of yogurt consumption on human health. In fact, frequent consumption of yogurt intake has been shown to improve risk factors for cardiovascular disease [10], to lower diabetes risk [11], enhance development of host immunity [12] and to lower the risk for dysbiosis and chronic kidney disease [13]. For instance, a study on the effects of *Lactobacilli*-containing yogurt on the composition of gut microbiota of healthy individuals who ingested a daily serving for 20 days, reported changes for two groups of bacteria (*Bacteroides* and *Prevotella*, and the *C. coccoides*-*E. rectalei* group) [14]. Another study showed that four weeks of probiotic yogurt consumption by healthy adults increased gut content of probiotic *Lactobacilli* and *Bifidobacteria* strains while not significantly altering microbial community structure [5]. Yogurt consumption by children infected by *Helicobacter pylori* was found to restore an optimal *Bifidobacterium*⁄*E. coli* ratio, decreased the *H. pylori* load, and altered serum immune response [15]. In addition, it was found that daily consumption of yogurt by healthy medical students increased the alpha diversity of the intestinal microbiome and was associated with reduced stress indicators [6]. Yet, there is limited information about the dynamics of gut microbial communities in individuals who consume yogurt regularly [16].

The aims of this study were to determine microbial community structure and composition using three different molecular methods after a high dosage of yogurt was administered to healthy human subjects. Terminal restriction fragment length polymorphisms (T-RFLP), cloning and Sanger sequencing, and next generation sequencing (NGS) were used in this study to characterize changes in microbial community structure, diversity, and phylogenetics of *Eubacteria* and also more specifically within the *Lactobacilli* and *Bifidobacteria*. We hypothesized that introducing a yogurt diet to human test subjects would: (i) alter microbial community structure; (ii) increase GI microbial diversity; and (iii) increase the abundance of probiotic bacteria.

## 2. Materials and Methods 

### 2.1. Enumeration and Confirmation of Probiotic Bacteria in Yogurt

Three commercially available brands of yogurt were tested for viability, following serial dilutions by inoculating onto selective media (LBS and BSM agar for *Lactobacillus* and *Bifidobacterium* respectively), and anaerobic incubations using GazPak Pouch Systems (BD technologies, Franklin Lakes, NJ, USA) at 37 °C for 48–72 h. Colony forming units (CFUs) were enumerated, and the yogurt with the highest CFUs per gram for both *Lactobacilli* (2.5 × 10^7^) and *Bifidobacteria* (1.8 × 10^7^) was selected for the study. The selected yogurt was a plain Greek yogurt (0% fat) with no fruit on the bottom. According to the manufacturer’s label, the active probiotic bacteria were listed as: *S. thermophilus*, *L. bulgaricus*, *L. acidophilus*, *L. casei* and *L. bifidus*. *L. bifidus* has long been reclassifided as the *Bifidobacterium* genus. 

### 2.2. Experimental Design and Sampling Collection

We followed appropriate approved protocols for human experimentation and all subjects gave written informed consent all of which were approved by the Institutional Review Board (IRB) at Youngstown State University (Youngstown, OH, USA). Volunteers did not receive any financial incentive to participate in the study. The study began with 8 healthy subject volunteers (2 females and 6 males) between the ages of 18 and 54. All participants who volunteered to consume yogurt for this study had no prior history of lactose intolerance (with the exception of the control subject) and stated that they had not taken antibiotics for six months prior to the study. None of the subjects took antibiotics during the length of the study. Neither dietary nor exercise logs were included in the study. Subjects were allowed to continue with their regular diet and activities during the length of the study. All subjects were healthy and remained healthy for 42 days. Eight test subjects were assigned letters A, B, C, E, F, G and H to keep anonymity. One subject, D, was included as a control since that individual had not consumed yogurt for several years, and agreed to not consume yogurt throughout the entire study. Two subjects E and H withdrew after 7 days because they did not like the high yogurt dietary regime. Prior to baseline (day 0), test subjects refrained from consuming any form of probiotics for a week. Starting at day 0, individuals consumed 250 g of the selected yogurt per day for 42 consecutive days. Yogurt (908 g) was provided to each subject every 3 to 4 days. Each participant provided fecal samples on days 0, 7, 14, 21, 28, 35 and 42. 

### 2.3. T-RFLP Analyses and Sanger Sequencing

Total DNA from subjects A, B, C, F, G and D (control) was extracted from fecal material using the QIAmp DNA Stool Isolation Kit (Qiagen, Valencia, CA, USA) according to the manufacturer’s instructions with minor modifications. A single DNA extraction per sample was performed. DNA extracts were stored at −20 °C until analysis. 

T-RFLP analysis was chosen because it is a reproducible, affordable, and sensitive method for monitoring changes in community structure [17]. Bacterial 16S rDNA were amplified using the primers 8F (5′-AGAGTTTGATCATGGCTCAG-3′) and 1492R (5′-GGCTACCTTGCCACGATTC-3′) [18]. Polymerase chain reaction (PCR) conditions are described in Table 1. For T-RFLP analysis, the forward primer (8F) was 5′ labeled with 6-carboxy-flourescein phosphoramidite (FAM). Each 25 µL PCR reaction contained: 0.5 µL of each primer (10 mM), 12.5 µL Go Taq Green Master Mix 1× (Promega, Madison, WI, USA), 2 µL of DNA, and 9.5 µL of molecular grade water. PCR product sizes were checked on 1% agarose gels and purified with the Wizard PCR preps DNA purification system (Promega, Madison, WI, USA) according to the manufacturer’s protocol. PCR products were digested using HaeIII and incubated at 37 °C for 16 h. After digestion, samples were purified using the Wizard® SV Gel and PCR Clean-Up system (Promega, Madison, WI, USA). Digested samples were sent to the Ohio State University Plant Microbe and Genomics Facility (Columbus, OH, USA) for fragment analysis using a 3730 DNA Analyzer (Applied Biosystems, Inc. Waltham, MA, USA). 

Cloning and Sanger sequencing was performed on DNA extractions of fecal material from subjects B, C, and D on days 0 and 42 to obtain 16S rDNA gene sequences for *Lactobacilli* and *Bifidobacteria* phylogenetics using primers Lac 1 (5′-AGCAGTAGGGAATCTTCCA-3′) and Lac 2 (5′-ATTYCACCGCTACACATG-3′) [19], and G-Bifid-F (5′-CTCCTGGAAACGGGTGG-3′) and G-Bifid-R (5′-CGTGTTCTTCCCGATATCTACA-3′) [20]. PCR conditions are shown in Table 1. Each PCR reaction contained: 0.2 μM of each primer, 10 μL of Go Taq Master Mix 1×, 2 μL of DNA, and 7.2 μL of molecular grade water. PCR products were purified using Wizard**®** SV and PCR Clean-Up System (Promega, Madison, WI, USA) and cloned into competent *Escherichia coli* cells using the Strataclone PCR cloning Kit (Agilent Technologies, Santa Clara, CA, USA) according to manufacturers’ protocols. Two sets of dilutions were plated for each sample. Transformed cells were plated on LB medium, containing 0.1 mg/mL of ampicillin and 4 μL of X-gal and incubated overnight at 37 °C. White recombinants were transferred to a 96-well plate containing LB medium, 0.1 mg/mL of ampicillin, and 10% glycerol, shaken for 24 h at 37 °C. The insert size of the colony was then determined by PCR amplification as described for *Lactobacillus* or *Bifidobacteria.* A total of 520 clones (273 from *Lactobacillus* and 243 for *Bifidobacteria*) were Sanger sequenced at the University of Kentucky Advanced Genetic Technologies Center, Lexington, Kentucky. *Bifidobacterium* and *Lactobacillus* 16S rDNA gene sequences were then checked for quality using the Vector NTI Software (Thermo Fisher Scientific, Waltham, MA, USA), manually selected, curated, and aligned using Clustal W. Evolutionary distance was constructed using MEGA version 5.0 [21] using the Neighbor-Joining method, and was calculated on the basis of Kimura’s two-parameter algorithm with 100 bootstrap resamplings. 

*Lactobacillus* genes were quantified in fecal DNA from subjects B, C and D for day 0 and day 42. Quantitative PCR (qPCR) was performed using the iQ™5 real-time detection system (Bio-Rad Inc., Hercules, CA, USA). The qPCR reaction was performed using a total volume of 25 μL of the following reagents: 12.5 μL of SYBR Green buffer (Qiagen, Valencia, CA, USA), 9.0 μL of RNase free water, 25 μM of primer, and 2 μL of DNA. The PCR conditions and primers are described in Table 1. Standard curves were constructed from purified genomic DNA from *Lactobacilli* isolated from probiotic pills using selective media (LBS).

Non-metric multidimensional scaling (NMDS) was used to compare T-RFLP restriction fragments (T-RFs) of gut microbial DNA from subjects A, B, C, F, G and D (control) at various time points. NMDS was chosen because it is an ordination technique that does not require linear variables (in contrast to principal-component analysis). There is also no need for specific distance measures (covariance), and it makes few assumptions about the data [22]. Statistical analyses were performed using IBM SPSS Statistics 20 for Windows (IBM Corp., Armonk, NY, USA). The Shannon Wiener diversity index (H’) was calculated using T-RFs to determine microbial diversity for each test subject at days 0, 7, 14, 21, 28, 35 and 42. 

### 2.4. Next Generation Sequencing

Total DNA was also used to amplify bacterial 16S rDNA, *Lactobacilli* and *Bifidobacteria* from test subjects (A, B, C, G and D) at days 0, 7, and 42 by NGS. Data for subject C on day 7 were missing, and all data for subject F were missing due to lack of fecal material. PCR reactions (Table 1) were performed in triplicate and followed PCR settings described previously. Concentrations of PCR products were determined by gel electrophoresis and then normalized according to band intensity. Subsequently, 20 μL of each normalized PCR product (*Lactobacilli*, *Bifidobacteria*, and *Eubacteria*) were pooled into a 96-well plate according to subject and sampling date, which was paired-end sequenced using an Illumina MiSeq platform (San Diego, CA, USA) and processed at the Case Western Reserve University Genomics Core Facilities (Cleveland, OH, USA).

Raw sequences were analyzed using the Quantitative Insight into Microbial Ecology (QIIME) pipeline [23]. Paired-end sequences were joined and removed from analysis if they were <200 nucleotides (nt), had a quality score < 25, contained < 6 ambiguous characters, and had a homopolymer length < 6. Additionally, the Usearch pipeline was implemented to check for chimeric sequences by the *de novo* and reference-based method with the SILVA ribosomal RNA gene database project (SILVA v. 119) [24]. Quality filtered sequences were clustered into operational taxonomic units (OTUs) using Usearch with a minimum identity of 97% [25]. The most abundant sequence from each OTU cluster was selected to represent that OTU. Representative sequences were aligned to the SILVA database using MUSCLE [26] and taxonomy was assigned using BLAST (2.2.22 version, National Center for Biotechnology Information, U.S. National Library of Medicine, Bethesda, MD, USA). Rarefaction was performed on all samples to standardize sequencing depth using a minimum subsampling depth (determined by the sample with the lowest number of sequences) at 2000 replicates. Alpha diversity was determined on rarefied sequences using Chao1 (richness) and Shannon Diversity (OTU-based diversity). Beta diversity was assessed by computing weighted UniFrac distances to compare microbial communities based on the fraction of unique branch lengths observed in pairs of communities for the phylogenetic tree and relative abundance for each microbial community. Jackknifing was incorporated by re-sampling 10 times with a sequencing depth of 20 sequences per sample. NMDS was performed to compare beta diversity among test subjects. Raw sequence data are available in the Sequence Read Archive at the National Center for Biotechnology Information under study PRJNA351747 with accession numbers SAMN05990293–990309.

## 3. Results

### 3.1. Viable Probiotic Bacteria in Dietary Yogurt 

Total viable *Lactobacilli* and *Bifidobacteria* consumed per day per subject were 6.3 × 10^9^ and 4.5 × 10^9^ CFUs respectively. This was based on consumption of 250 g of yogurt in which we detected 2.5 × 10^7^ and 1.8 × 10^7^ CFUs per gram of *Lactobacilli* and *Bifidobacteria* respectively.

### 3.2. GI Bacterial Communities

#### 3.2.1. Variation and Diversity based on Analysis of T-RFLPs 

**Figure 1:**
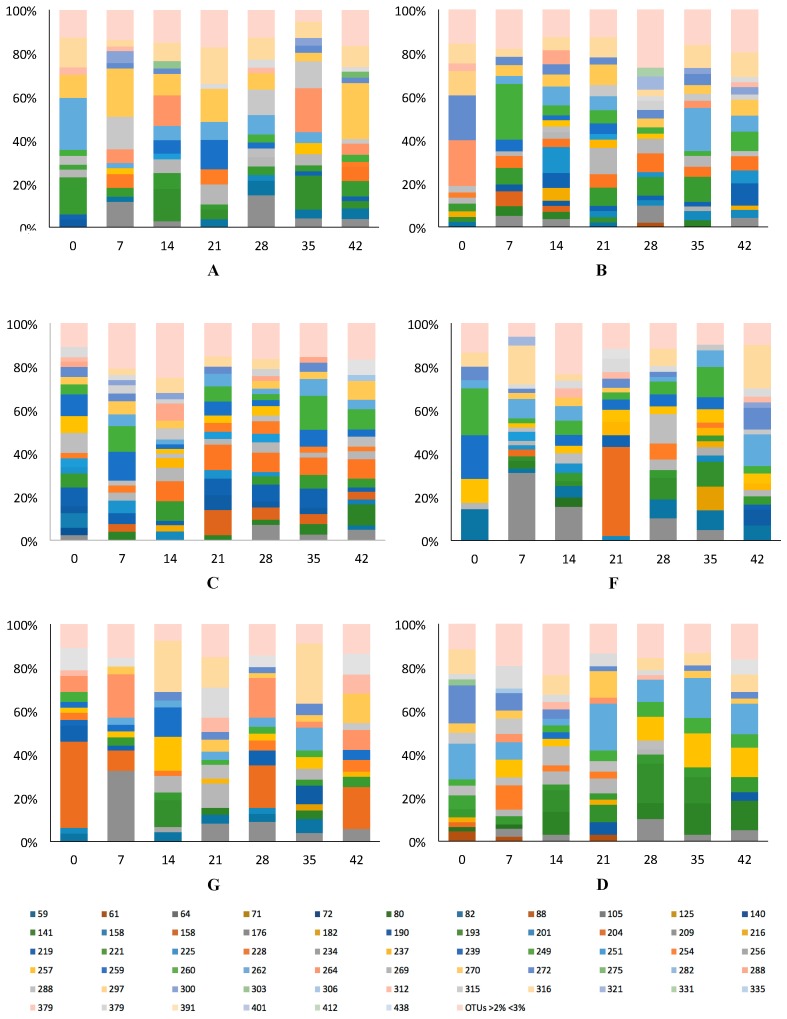
Bacterial community composition in gastrointestinal (GI) tract for test subjects A, B, C, F, G and D (control) on days 0, 7, 14, 21, 28, 35 and 42. Figure shows terminal restriction fragments [T-RFs]-bp that contributed with more than 2% to the total relative abundance.

**Figure 2:**
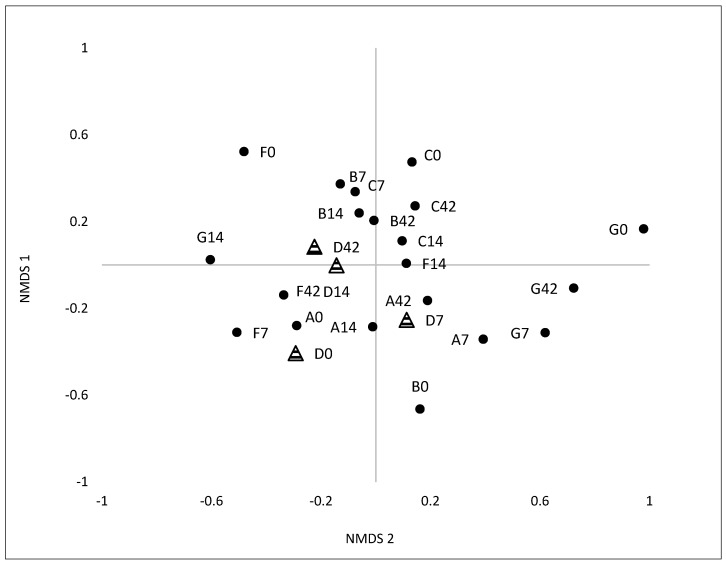
Non-metric multidimensional scaling (NMDS) showing distribution of microbial communities (based on T-RFs of *Eubacteria* 16S rDNA) for test subjects A, B, C, F, G (circles) and control subject D (triangles). For clarity and comparison data shown is only for days 0, 7, 14, and 42 (excluded day 21, 28 and 35).

A total of 61 *Eubacteria* bacterial terminal restriction fragments (T-RFs), ranging from 59 to 438 bp in length, were detected from collected fecal samples (Figure 1). The number of T-RFs showed high variability throughout the length of the study. Bacterial community profiles were dominated by a number of T-RFs that contributed to higher relative abundance. Only a small percentage (ranging from 7% to 20%) contributed to less than 3% to the total relative abundance (Table 2). Analysis of T-RF profiles by NMDS differentiated the GI microbial samples according to sampling time (Figure 2). T-RFs at day 0 were the furthest away from the center of the axis for test subjects A, B and F, but they begin to differentiate at day 7. By day 14 of yogurt consumption, microbial communities begin to cluster together, indicating microbial communities of similar composition (Figure 2). Test subject D (control) also differentiated over time, but not as drastically as the experimental group. 

The Shannon Wiener diversity index (H’) based on GI *Eubacteria* T-RFs (Table 3) was highest for test subject C for all time points except for day 28. Diversity increased from day 0 to day 14. However, after day 14 microbial diversity appeared to plateau and stay in a state of flux throughout the remainder of the study (Table 3). It should be noted that all test subjects had higher H’ values at each time point when compared to day 0, except for test subject A and control subject D. Test subjects G and F had the lowest H’ at day 0 in comparison to all other test subjects and also showed the greatest increase in H’ values for all time points, when compared to day 0. However, the average diversity from day 0 and day 42 for all subjects consuming yogurt was not statistically significantly different (*p* > 0.05). There was no significant change in gut *Eubacteria* diversity associated with yogurt consumption. 

#### 3.2.2. Variation and Diversity based on NGS 

**Figure 3:**
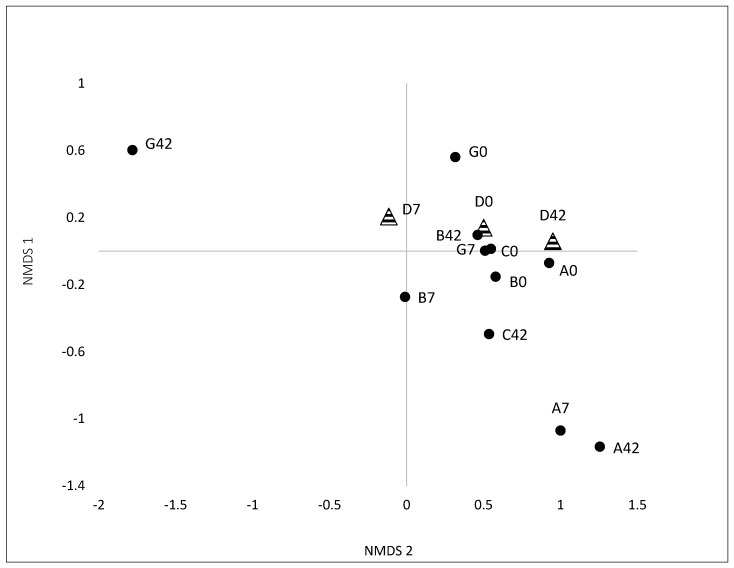
Non-metric multidimensional scaling (NMDS) showing distribution of microbial communities (rarefied data based on NGS of the *Eubacteria* 16S rDNA) for test subjects A, B, C, G (circles) and control subject D (triangles) on days 0, 7, and 42.

Yogurt-consuming subjects A, B, C, and G, and control subject D, were chosen to determine community structure and bacterial diversity within fecal samples from day 0, prior to yogurt consumption, day 7 and after 42 days of yogurt consumption using Illumina platform for NGS. Subject F was not included because of insufficient fecal material provided. The forward and reverse primers chosen to amplify *Eubacteria* rDNA for NGS did not provide overlapping sequences. Thus only data generated from the forward primer (R1), were chosen for analyses since there were more quality sequences (8129) than from the reverse primer R2 (6483). NMDS of the *Eubacteria* NGS R1 data (Figure 3) indicated that microbial communities within individual subjects diverged from day 0, becoming more dissimilar upon yogurt consumption with time at day 7 and day 42. Interestingly, this shift was also observed for the test control subject. There was no clear clustering of data by subject or by sampling date.

The Shannon Wiener diversity index (from subjects A, B, C, G and D) based on *Eubacterial* R1 NGS data ranged from 5.2 to 5.4 on both day 0 and day 42 in all subjects, with only one outlier, subject G, whose diversity was much lower (3.5) at day 42 (Table 4). Similarly, the Chao1 index (based on the same data as above) showed the same trends, ranging from 89 to 100 for the three yogurt consuming subjects on day 0, which on day 42 spread out to include values of 103 (subject B), 95 (subject C) and the lowest value 49 (subject G), while the Chao1 index for control subject D increased from 96 to 119 on day 42. Both indexes were not statistically significantly different from day 0 to day 42 (*p* > 0.05).

**Figure 4:**
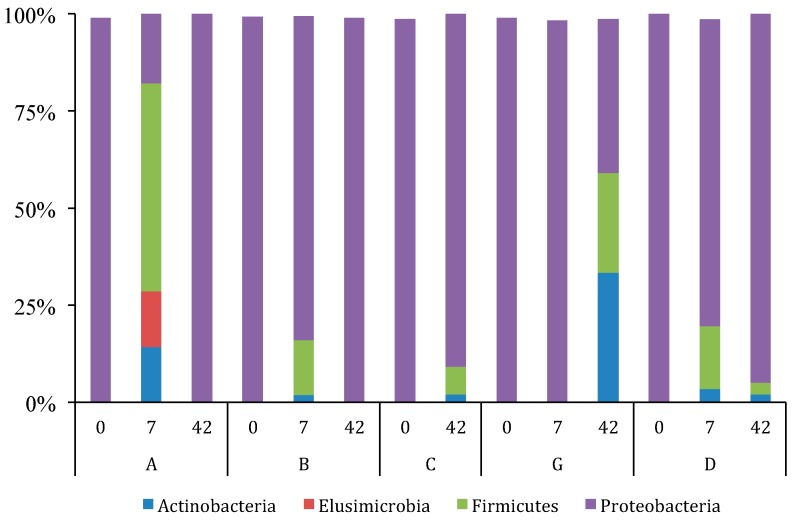
Relative abundance of *Eubacteria* rDNA sequences based on NGS for test subjects A, B, C, G and D (control) on days 0, 7, and 42. Test subject C had a missing data point on day 7. Figure shows relative abundance of taxa that contributed more than 2% to the total relative abundance.

Analyses of the relative abundance of bacteria (Figure 4) using all available data from treatment subjects A, B, C, G and D between 0 and 42 days of yogurt consumption revealed that on day 0, 99% of the taxa detected were *Proteobacteria* (all of which are Gram (−) bacteria). By day 7 *Proteobacteria* decreased to 79% with increased Gram (+) bacteria, *Firmicutes* (15%, which include *Lactobacillus*), *Actinobacteria* (4%, which include *Bifidobacteria*), and *Elusimicrobia* (3%). By day 42 *Proteobacteria* further decreased to 68%, while *Firmicutes* and *Actinobacteria* increased to 17% and 14% respectively. Among the *Proteobacteria*, the majority of sequences were members of the *Gammaproteobacteria* related to *Escherichia-Shigella* and *Pantoea* species, which were present in all subjects at different sampling dates. Interestingly, the majority of species observed were recorded for day 7 for all consuming yogurt subjects. Members of both *Firmicutes* and *Proteobacteria* were each both represented by four phyla that fluctuated in relative abundance throughout the study (data not shown).

#### 3.2.3. *Lactobacilli and Bifidobacteria*: Sanger Analyses

Yogurt consuming subjects B and C and non-yogurt consuming control subject D were selected to determine phylogenetic distribution of gut *Bifidobacteria* and *Lactobacilli* from day 0 and day 42. A total of 263 *Bifidobacteria* gene sequences (67, 120 and 76 for subjects B, C, and D, respectively) and 146 *Lactobacillus* gene sequences (48, 39 and 59 for test subjects B, C and D, respectively) were analyzed. The most even phylogenetic distribution within the *Bifidobacteria* was found in subject C (Table 5) at day 0 in which the four detected species were all present with *B. bifidum* as the most abundant (44%). At day 42, the most abundant phyla within subject C was *B. adolescentis,* which increased from 24% to 62% at the expense of *B. bifidum*, which dropped from 44% to 17%, and *B. pseudocatenalatum,* which dropped from 10% to 0%. Subject B showed the least diversity within the *Bifidobacteria*, where *B. pseudocaenulatum* and *B. longum* accounted initially for 81% and 19% of the *Bifidobacteria* respectively. At day 42, in subject B, all of the *Bifidobacteria* detected were *B. longum*. Unexpectedly subject D, who had never consumed yogurt, showed major changes in relative abundance for three of the major *Bifidobacteria* species from day 0 to day 42: *B. longum* (47% to 0%), *B. pseudocatenalatum* (9% to 51%), and *B. bifidum* (0% to 8%), while *B. adolescentis* remained within 3% (44% to 41%).

Overall there were more *Lactobacilli* than *Bifidobacteria* species (15 vs. 5) detected within the three subjects. *L. casei* was the most abundant *Lactobacteria* found within all three subjects and was only absent in subject D on day 42. *L. casei*, and *L. rhamnosus* were the only *Lactobacilli* that increased in relative abundance in subjects B and C following yogurt consumption. Most unexpectedly, both the most *Lactobacillus* species detected (9) and the highest *Lactobacillus* species diversity were found in control subject D at day 0. These nine species were evenly distributed (Table 5) with *L. rhamnosus*, *P. stilesii* and *L. casei* being the most abundant. At day 42 all species were detected, except one. Only *L. gasseri* was detected on both dates and it showed a major increase from 8% to a prominent 69%. Subject D also had the most unique species detected (Table 5). Subject C showed intermediate *Lactobacillus* species diversity with 5 species detected on day 0, and 4 species detected on day 42. Only *L. casei* and *L. ruminis* were detected on both days, increasing from 10% to 26% and 5% to 22% respectively. *L. gasseri*, *L. crispatus*, and *P. stilesii* were relatively abundant at day 0 (45%, 20% and 20%, respectively) but were not detected at day 42. The opposite trend was found for *L. rhamnosus* and *L. reuteri*, which were both not detected at day 0 but equally relatively abundant on day 42 (26%). *L. reuteri* was the only *Lactobacilli* uniquely detected in subject C on a single date. Subject B had the fewest *Lactobacilli* species detected (3) which corresponded with the lowest *Lactobacillus* species diversity (only 2 species were detected on each day). *L. casei* was the most abundant on both sample dates and increased from 73% to 95% on day 0 and 42 respectively. *L. rhamnosus* was only detected on day 42 with a relative abundance of 5%. *L. iners* was the single uniquely detected *Lactobacilli* and was only detected in Subject B at day 0 with a relative abundance of 27%.

#### 3.2.4. *Lactobacilli* and *Bifidobacteria*: NGS

**Figure 5:**
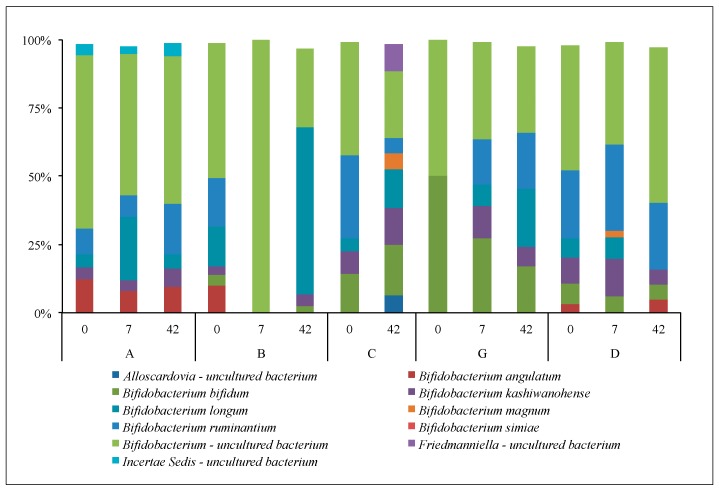
Relative abundance of *Bifidobacteria* species recovered from next generation sequencing (NGS) for test subjects A, B, C, G and D (control) on days 0, 7 and 42. Test subject C had a missing data point on day 7. Figure shows relative abundance of *Bifidobacteria* that contributed with more than 2% to the total relative abundance.

NGS phylogenetic data, based on 81,566 *Bifidobacteria* sequences after filtering, indicated that there were 8 identified species of *Bifidobacteria* and 3 other related species detected at the 1% level or more; however most of these were not relatively abundant (Figure 5). There was no particular *Bifidobacterium* species that consistently increased in relative abundance following yogurt consumption across subjects. Unidentified species of *Bifidobacterium* were relatively abundant (ranging from 25% to 64%) compared to identified species of *Bifidobacterium*. *B. longum* (ranging from 4% to 61%) and *B. ruminatium* (ranging from 2% to 31%) were the relatively most abundant of the identified *Bifidobacterium* species in all subjects, but not on all sample dates. *B. bifidum* (1% to 27%) followed by *B. angulatum* (<1% to 13%) were also present, but not in all subjects.

**Figure 6:**
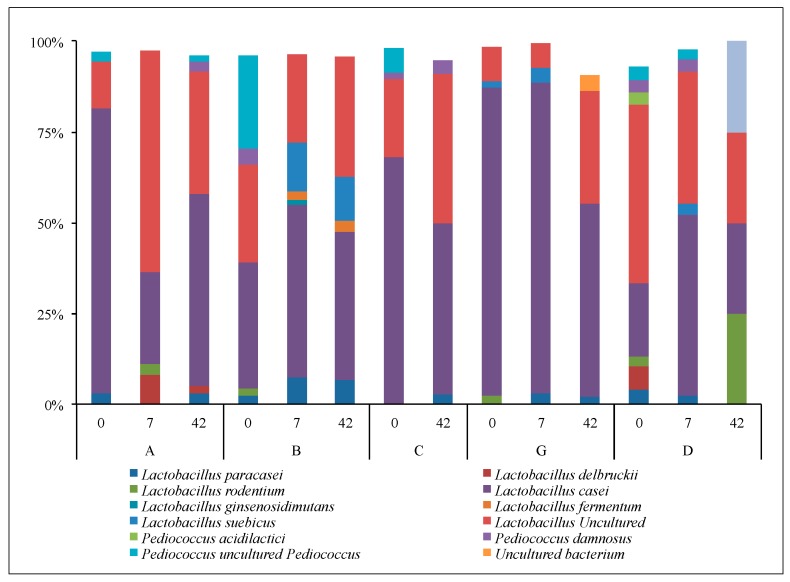
Relative abundance of Lactobacillus species recovered from NGS for test subjects A, B, C, G and D (control) on days 0, 7, and 42. Test subject C had a missing data point at day 7. Figure shows relative abundance of Lactobacillus that contributed with more than 2% to the total relative abundance.

There were 6483 *Lactobacilli* phylogenetic sequences after filtering data, which were represented by 8 *Lactobacilli* species and 4 related species related detected at 1% relative abundance or greater within at least one subject at least once (Figure 6). Only a handful of these were relatively abundant in most subjects at most sample dates. The most relatively abundant taxon was *L. casei* followed by uncultured *Lactobacillus*, which together made up 80% to 90% of the detected *Lactobacilli* within all subjects at most sampling dates. The remaining 10% to 20% of the *Lactobacilli* mainly consisted of *L. paracasei*, *L delbuckii*, and *L. suebicus*. None of the *Lactobacilli* taxa consistently increased upon yogurt consumption. 

### 3.3. Quantitative PCR of Lactobacillus 16s rDNA Genes

Quantification of *Lactobacillus* 16S rDNA genes revealed that there was a relative increase in number of *Lactobacillus* bacteria after 42 days of yogurt consumption for subject B (from 7 × 10^−5^ to 8 × 10^−1^ μM of rDNA). In contrast, *Lactobacillus*, which was initially relatively high, remained within an order of magnitude in test subject C (from 2 × 10^−1^ to 6 × 10^−2^ μM). The control subject D showed the lowest difference in number of *Lactobacillus* bacteria (from 6 × 10^−4^ to 2 × 10^−4^ μM).

## 4. Discussion

This is the first study to report weekly monitoring of the human gut microbiota following dietary yogurt consumption using a combination of molecular techniques, and might be the first to apply molecular techniques to examine the short term effects of dietary yogurt rather than more concentrated dietary applications of probiotics. This study included weekly sampling to determine effects of daily yogurt consumption on the composition and diversity of the gut microbiome of five healthy human subjects. A control subject, who was strongly lactose intolerant and had never consumed yogurt was included to determine semi-quantitative patterns of microbial communities compared to subjects consuming yogurt. Many earlier studies utilizing yogurt typically lasted 10 to 20 days and used either culturing techniques or identified certain microorganisms using probe markers [14,27]. 

In this study, we examined the temporal changes of GI microbial communities in test subjects that consumed 250 g of yogurt per day for 42 consecutive days. The amount of probiotic bacteria consumed (CFU per day) in this study (~10^9^) was on than the low end of the range (10^8^–10^12^) of multiple probiotic studies previously described [28]. However, other recent studies focused on probiotics delivered as pill or liquid form, and did not include dietary probiotic yogurt. Cormier et al. [29], reported that the average yogurt consumption was 82 g per day (excluding those that do not consume yogurt), and that approximately less than 3% of the population consumes 250 g of yogurt or more per day. Similarly, Margolis et al. [30] reported that for North American women the median intake of yogurt (of those who consumed yogurt) was one-half serving per week (~75 g). Wang et al. [31] reported that (of those who consume yogurt) the average weekly yogurt consumption is 341 grams per week for North Americans. Ortinau et al. [32] found positive effects attributed to yogurt in healthy women in the USA that consumed 3 to 4 times a week 170 g of Greek yogurt as an afternoon snack. In contrast, people in Europe and Australia tended to eat more yogurt. For example, Ivey et al. [33] reported yogurt intake as low (<100 g/day), moderate (100 to <200 g/day) and high (>200 g/day) for Australian women. Sayon-Orea et al. [34] included a range of yogurt consumption of 0–250 g per week as baseline, and a high dose as >875 gram per week for people from Spain. The amount of yogurt consumed by volunteers in this present study was already much greater than the amount normally consumed by the general population in the USA but is within the high end of the range found in Europe and Australia. Thus, based on the literature the dose of dietary yogurt (1750 grams/week) was chosen to increase chances of seeing a probiotic effect. 

Total DNA extracted from fecal samples was analyzed by T-RFLPs, cloning/Sanger sequencing, and NGS/Illumina MiSeq, three DNA based approaches independent from culturing. Earlier research relied on culture-dependent methods, which have provided useful information about intestinal microbiota, though only about 20% of GI bacteria are actually cultivable [35]. Molecular techniques are now standard for analyzing the human microbiome [36]. Thus, we chose a multi approach that combined classical fingerprinting techniques with state-of-the-art tools. 

NMDS based on T-RFLPs data showed a trend in which the microbial community composition begins to change by 7 days of yogurt consumption. At days 14 and 42, the microbial communities clustered together for individuals consuming yogurt. This clustering suggested that the microbial communities were becoming structurally more similar after 14 days of yogurt consumption and maintained that similarity for the remainder of the study. Previous research by David et al. [37] has shown similar short-term alterations of the gut microbiome when a diet is changed between an animal or plant based diet. In this present study, clustering of GI microbial communities might have been the result of the test subjects sharing similar dietary habits (i.e., yogurt consumption). Omar et al. [38] had also observed a significant change in the gut microbiome (i.e., decreased *Clostridia* cluster V) due to introduction of dietary probiotic *Lactobacillus* species (10^9^ per day). This contrasts with another previous study, which indicated that dietary yogurt consumption by healthy human subjects increased the target probiotic strains but without corresponding changes in microbial T-RFLP profiles within the fecal material beyond inter-subject variation [5]. NMDS analyses of *Eubacteria* NGS data showed microbial community divergence over time following yogurt consumption. These contradictory results from two different methods (T-RFLPs and NGS) are not entirely unexpected. Using a known mock bacterial community consisting of 20 bacteria species, Fouhy et al. [39] demonstrated that the choice of primers and sequencing methods gave extreme variable results. 

The Shannon-Wiener index (H’), a commonly used estimate of ecological diversity [40] was used to quantify changes in gut microbial diversity (recovered from fecal material) based on terminal restriction fragments (T-RFs) and on NGS data of universal bacteria primers after the consumption of yogurt. The Shannon index takes into account both the number of bacterial groups (richness), the abundance (evenness) of each group, and the changes in rare groups that maybe present within an ecosystem [41]. This current study indicated no significant change in gut microbial diversity based on T-RFs when comparing day 0 and day 42, i.e., before and after 42 days of yogurt consumption, although the average value slightly increased from H’ = 2.6 (±0.3, n = 5) to H’ = 2.8 (±0.3, n = 5). However, the first two weeks of yogurt consumption did show a general trend towards greater diversity, followed by a plateau and then a drop by day 42. This may have been due to dietary disturbance followed by a dynamic readjustment of species present. Similarly, diversity (H’) was also relatively unchanged when comparing day 0 and following 42 days of yogurt consumption using NGS, though the values were much higher (~4.1). The very different Shannon index values based on two different approaches were likely due to the differences in techniques; although the targeted DNA region was the same, NGS had more sequence data than T-RFs (by several orders of magnitude). A more comprehensive study with larger number of volunteer subjects would be needed to establish clearer trends of GI diversity. 

There is little consensus in expected phylogenetic distribution and community composition of gut bacteria within the human colon. Sankar et al. [42] reported the distribution as *Proteobacteria* (~45%), *Firmicutes* (~32%), *Bacteriodetes* (~22%), and a small percentage of *Actinobacteria* making most of the remainder. This differs from a study showing the distribution of human gut bacteria detected in elderly people as largely *Firmicutes* (~50%), *Bacteroides* (~35%), and *Proteobacteria* (~8%) [25]. In our study the relative abundance of *Proteobacteria* was initially 99% of the detected bacteria taxa. By day 7, *Proteobacteria* decreased to 79% and 68% by day 42 respectively, with increased abundance of *Firmicutes* and *Actinobacter*. Kim et al. [43] also found that probiotic consumption altered the relatively robust gut microbiome (*Bacteriodetes*), but only about 3% of the OTUs detected changed by an order of magnitude. Surprisingly, we did not detect a major proportion of *Bacteriodetes*. This could have been due to the lack of primer specificity used for NGS. Unexpectedly, *Elusimicrobia* made up 14% of the OTUs detected in one subject at day 7, which is the only value that contributes to the *Elusimicrobia* average abundance of 3% for that date. This may be due to a subject that originated outside of North America with some rarely reported microbiome taxa.

Although the microbial community composition of *Bifidobacteria* did shift after 42 days of yogurt intake, we did not observe any particular trend in changes of *Bifidobacteria* overtime. However, clone library sequencing of *Lactobacilli* bacteria indicated that there were increased abundance of *L. casei* in the two subjects who consumed yogurt (containing *L. casei*) and not in the control subject. This is the clearest indication that the probiotic *Lactobacilli* survived the GI passage. These data were somewhat supported by qPCR analysis which showed greatly increased *Lactobacillus* for one (but not both) of the test subjects that consumed yogurt daily. Sanger sequencing produces more accurate sequence information than NGS, having 0.01 less error rate than NGS [44]. In this study Sanger sequencing provided more reliable data of relative abundance of *L. casei* than NGS.

Overall, we observed that after 42 days of yogurt consumption, there was a fluctuation in diversity and a change in GI composition, suggesting that introducing dietary yogurt influenced the gut microbiome. Statistical analyses indicated that microbial communities had a shift in community composition by becoming either more similar or dissimilar overtime depending on the method of analyses. These findings need to be taken cautiously since other external factors (not measured in the present study) might have influenced community composition. We did not follow diet regimens and did not restrict any food consumption. It could also be argued that 1-week abstention from any form of probiotics might have been insufficient to wash-out the microbial community to a baseline and therefore lessened potential effects of yogurt on the GI community. However, an effective probiotic abstention period would also alter the GI community and could be considered an artifact of the study since subjects had been eating yogurt as part of their diet. 

Limitations of this study might have influenced the overall study findings. First, we had a small number of participants willing to consume high doses of plain Greek yogurt daily with no fruit on the bottom. A number of potential subjects did not want to provide fecal samples due to the “ick” factor, and some subjects who were included in the study did not provide sufficient fecal material for all analyses (missing data). Some analyses were not performed on all subjects due to time and budgetary constraints. Lastly, we recognize that our results may not be powerful enough to predict effects of dietary yogurt on GI communities. Further research involving a large number of subjects and equal number of controls could provide more statistical robustness of the effect of yogurt on microbial communities. 

## 5. Conclusions 

Analyses of the GI microbial community composition and diversity revealed no significant changes during consumption of yogurt. Regular monitoring indicated that yogurt intake appeared to have an effect on *Lactobacilli*. The high dose of yogurt appeared to slightly increase microbial diversity in some individuals. However, more research is needed to determine if dietary yogurt intake could have an effect on GI microbial community structure and diversity.

## Figures and Tables

**Table 1:** Primers for 16S rDNA and Polymerase chain reaction (PCR) settings used in the present study.

Target	Primers	Sequence (5'-3')	PCR Settings	Ref.	Used for
*Eubacteria*	8F 1492R (~1400 bp)	AGAGTTTGATCATGGCTCAG GGCTACCTTGCCACGACTTC	94 °C 3 min 94 °C 30 s 53 °C 1 min (35×) 72 °C 90 s 72 °C 7 min	[18]	T-RFLP, NGS
*Lactobacillus*	Lac 1 Lac 2 (380 bp)	AGCAGTAGGGAATCTTCCA ATTYCACCGCTACACATG	94°C 3 min 94°C 30 s 53°C 1 min (35×) 68°C 1 min 68°C 7 min	[19]	NGS, Sanger, qPCR
*Bifidobacteria*	G-Bifid-F G-Bifid-R (549–563 bp)	CTCCTGGAAACGGGTGG CGTGTTCTTCCCGATATCTACA	95°C 5 min 95°C 30 s 55°C 20 s (30×) 72°C 30 s 72°C 5 min	[20]	NGS, Sanger, qPCR

**Table 2:** Number of terminal restriction fragments (T-RFs) that contributed with more than 2% to the total relative abundance for test subjects A, B C, F and G, and control subject D at days 0, 7, 14, 21, 28, 35 and 42.

Days	Subjects						
	A	B	C	F	G	D	
0	11	12	18	8	12	15	
7	13	13	16	17	10	15	
14	12	18	17	15	11	15	
21	10	16	15	12	13	14	
28	14	17	20	14	13	12	
35	14	15	15	15	14	10	
42	15	15	17	15	10	11	

**Table 3:** Shannon Weiner (H’) diversity (based on T-RFLP data) for all test subjects (A, B, C, F and G) and control subject (D) measured at each sampling date. Bold values indicate the highest indices measured at each time point during the study.

Day	A	B	C	F	G	D
0	2.49	2.63	**3.06**	2.25	2.32	2.77
7	2.72	2.86	**3.18**	2.44	2.36	2.98
14	2.78	3.11	**3.33**	3.18	2.39	3.07
21	2.64	3.00	**3.02**	2.35	2.86	2.80
28	2.84	**3.38**	3.32	2.87	2.71	2.83
35	2.56	2.91	**3.00**	2.83	2.60	2.48
42	2.38	3.11	**3.13**	2.73	2.67	2.99

**Table 4:** Shannon (H’) and Chao1 diversity index values based on the number of operational taxonomical units recovered from *Eubacteria* NGS for test subjects A, B, C, G and D (control) at days 0 and 42.

*Eubacteria*				
Subject	H’		Chao1	
	Day 0	Day 42	Day 0	Day 42
A	5.35	NA	89.37	NA
B	5.33	5.36	95.77	103.19
C	5.33	5.25	94.87	95.43
G	5.33	3.48	99.53	48.63
D	5.31	5.41	96.50	119.04

**Table 5:** Relative abundance of *Lactobacilli* and *Bifidobacteria* (based on Sanger sequencing analyses) for two subjects consuming yogurt B and C, and for control subject D on day 0 and day 42.

Subject	Day 0		Day 42	
	*Bifidobacterium*	*Lactobacillus*	*Bifidobacterium*	*Lactobacillus*
B	*B. longum* 19% *B. pseudocatenulatum* 81%	*L. iners* 27% *L. casei* 73%	*B. longum 100%*	*L. casei* 95% *L. rhamnosus* 5%
C	*B. longum* 21% *B. bifidum* 44% *B. adolescentis* 24% *B. pseudocatenulatum* 10%	*L. gasseri* 45% *L. crispatus* 20% *L. casei* 10% *Pediococcus stilesii* 20%	*B. longum* 17% *B. bifidum* 17% *B. adolescentis* 62% *Alloscardovia omnicolens* 4%	*L. casei* 26% *L. rhamnosus* 26% *L. reuteri* 26% *L. ruminis* 22%
D	*B. longum 47%* *B. adolescentis 44%* *B. pseudocatenulatum 9%*	*L. gasseri* 8% *L. crispatus* 4% *L. sakei* 4% *L. casei* 13% *L. rhamnosus* 29% *L. plantarum* 8% *Dolosigranulum pigrum* 4% *Granulicatella adiacens* 4% *Pediococcus stilesii* 25%	*B. bifidum* 8% *B. adolescentis* 41% *B. pseudocatenulatum* 51%	*L. gasseri* 69% *L. delbrueckii* 9% *L. homohiochii* 6% *Weissella paramesenteroides* 17%
